# Organ-Specific, Fibroblast-Derived Matrix as a Tool for Studying Breast Cancer Metastasis

**DOI:** 10.3390/cancers13133331

**Published:** 2021-07-02

**Authors:** Adina R. D. Jensen, Edward R. Horton, Lene H. Blicher, Elin J. Pietras, Cornelia Steinhauer, Raphael Reuten, Erwin M. Schoof, Janine T. Erler

**Affiliations:** 1 Biotech Research and Innovation Centre (BRIC), University of Copenhagen (UCPH), 2200 Copenhagen, Denmark; adina.jensen@bric.ku.dk (A.R.D.J.); ewho@novonordisk.com (E.R.H.); elin.pietras@bric.ku.dk (E.J.P.); cornelia.steinhauer@bric.ku.dk (C.S.); raphael.reuten@bric.ku.dk (R.R.); 2 Department of Biotechnology and Biomedicine, Technical University of Denmark, 2800 Lyngby, Denmark; lench@bio.dtu.dk (L.H.B.); erws@dtu.dk (E.M.S.)

**Keywords:** fibroblast-derived matrix, cancer, drug screening

## Abstract

**Simple Summary:**

Cancer in the breast often spreads to other parts of the body, such as the lungs, which leads to poor outcomes for patients, as there are few effective treatments. Within organs such as the lungs, cancer cells are surrounded by a scaffold, made of proteins, which helps keeps the organs’ structure and maintains their function. This scaffold is produced by cells called fibroblasts, and we can reproduce this in the lab. We aim to investigate how cancer cells interact with the protein scaffold from different organs, where breast cancer cells spread to. This study hopes to reveal how breast cancer reacts to different organ environments and use this method to perform large-scale drug screening. Importantly, this study has shown that drug testing of breast cancer cells within a more physiological context, as opposed to testing on plastic, can lead to increased identification of targets to treat breast cancer.

**Abstract:**

During the metastatic process, breast cancer cells must come into contact with the extra-cellular matrix (ECM) at every step. The ECM provides both structural support and biochemical cues, and cell–ECM interactions can lead to changes in drug response. Here, we used fibroblast-derived ECM (FDM) to perform high throughput drug screening of 4T1 breast cancer cells on metastatic organ ECM (lung), and we see that drug response differs from treatment on plastic. The FDMs that we can produce from different organs are abundant in and contains a complex mixture of ECM proteins. We also show differences in ECM composition between the primary site and secondary organ sites. Furthermore, we show that global kinase signalling of 4T1 cells on the ECM is relatively unchanged between organs, while changes in signalling compared to plastic are significant. Our study highlights the importance of context when testing drug response in vitro, showing that consideration of the ECM is critically important.

## 1. Introduction

Metastatic disease contributes to 66–90% of cancer deaths [1,2]; therefore, understanding the complex interactions of cancer cells and their microenvironment at the primary and metastatic sites will be vital in providing effective treatment. The process of metastasis is multi-step, complex, and significantly driven by the microenvironment. Cells are embedded in a 3D scaffold within tissues known as the extra-cellular matrix (ECM) [3]. Cell-ECM interactions play a fundamental role in the proper regulation of cell behaviour and homeostasis, especially in response to environmental changes and stresses [4]. In particular, cancer-cell–ECM interactions are critical for metastasis since cancer cells must interact with ECM at every stage of the metastatic cascade, eventually attaching to the ECM of the secondary organ to initiate growth; the so-called seed (cancer cells) and soil (organ ECM) hypothesis [5].

The tumour microenvironment is comprised of many different cell types, which together interact and regulate the ECM. Fibroblasts are highly involved in the production and remodelling of the ECM in vivo, and during normal development and physiology, these cells produce mainly connective tissue ECM [6,7]. Specifically, ECM at distinct organ niches is different in composition, structure, and biophysical properties, often related to function in normal physiology. The ECM composition of the lung, for example, is rich in fibrillar collagens and elastin, which are important to allow the necessary ‘recoil’ [8]. At the primary site in breast cancer, the tumour’s ECM is often stiffer [9] and characterised by an increase of collagen I remodelling and deposition, which facilitates local invasion due to increased linearisation of fibres [10]. During tumorigenesis, resident fibroblasts become ‘activated’, similar to when they are activated during normal wound healing [11]. The global composition of the ECM has been catalogued using both in silico and biophysical approaches leading to what is known as the ‘matrisome’; a database containing 1056 genes describing 278 ‘core’ ECM proteins [12]. Recently it has become possible to produce native ECM from fibroblasts in vitro and use the ECM that they produce in cancer investigations [13].

Cells interact with the ECM via integrins and glycoproteins, which in turn transduce signals internally, leading to the activation of intracellular kinases [4]. Kinase activation via protein phosphorylation within cells is a key post-translational modification that is involved in the regulation of intracellular signalling, which leads to the induction of gene expression, metabolic changes, proliferation, differentiation, and membrane transport [14]. Over 500 kinases are identified in the human genome [15] and are divided into two categories: tyrosine kinases (PTKs) and serine/threonine kinases (STKs) [16]. Tyrosine kinases transfer a phosphate molecule from ATP to the tyrosine residue [17]. Aberrant activation of receptor tyrosine kinases has been described in carcinogenesis, examples of which include the overexpression of human epidermal growth factor receptor (EFGR) and platelet-derived growth factor receptor α/β (PDGFR α/β) [18,19]. There are currently (as of September 2020) 52 receptor tyrosine kinase inhibitors currently approved by the US-FDA, 46 of which are used in the treatment of neoplastic diseases [20], indicating an increased use and focus within cancer treatment.

There has been an increasing number of in vitro approaches to anti-cancer drug testing, often balancing increasing complexity that better represents tumours and the ability to screen at a high throughput level. These approaches range from simple viability assays, where cancer cells on plastic are treated with a prospective compound, and cytotoxicity is measured [21], to complex patient-derived 3D multicellular cultures [22]. Issues with cancer cells treated on a two-dimensional monolayer are that they often do not reflect complex three-dimensional tissue architecture or cell–ECM interactions [23]. However, with increasing complexity and dimensionality, issues with maintenance and control during testing lead to decreased screening capacity. Therefore, it is crucial to continue to investigate new in vitro experimental approaches that consider the importance of the tumour microenvironment and especially the ECM in anti-cancer drug testing.

Many breast cancer types have a poorer clinical outcome if the disease has progressed to stage IV (metastatic disease) [24,25]. Breast cancer typically metastasises to the lymph nodes, lungs, liver, bones, and brain, and currently, treatment options for distant metastasis include surgery, radiation, and a small number of chemotherapeutic agents [26]. However, these treatments often have limited efficacy and impact on patient survival.

In this study, we have focused on breast cancer and used the highly metastatic 4T1 mouse mammary carcinoma cell line (and 67NR non-metastatic counterpart) [27], which readily metastasises to lymph nodes, lungs, and liver, and most closely resembles the basal/TNBC (triple-negative breast cancer) breast cancer subtype [28]. Here, we have used fibroblasts isolated from mouse lymph node, lung, and mammary gland tissue to generate 3D fibroblast-derived matrices (FDMs) that closely resemble in vivo mesenchymal matrices [13,29]. We can use these FDMs to expose breast cancer cells to different ECM organ environments (primary and metastatic sites), analyse induced global kinase signalling in the cancer cells, the proliferation of cancer cells, and importantly, conduct a large-scale drug screening.

In this study, we show that despite differences in FDM composition between primary and metastatic sites, global kinase signalling induced in breast cancer cells on FDMs remains relatively unchanged between different organ ECMs. We observe that when exposed to the ECM, 4T1 breast cancer cells induce different global kinase signalling signatures comparative to plastic, which correlates with drug response. Importantly, we reveal that the drug response differs significantly between cancer cells treated on the FDMs and plastic, calling into question the relevance of using plastic for large-scale drug screening in the pre-clinical setting. We show that while the changes in lung/mammary FDM we produce overlap nicely with in vivo protein composition of the lung vs. mammary decellularised organs, we can elucidate similar responses in kinase signalling and drug response when breast cancer cells are exposed to the ‘simpler’ commercially available matrices. This paves the way for simple improvements in drug testing that considers the important role of the ECM.

## 2. Materials and Methods

### 2.1. Cell Lines and Cell Culture

4T1 and 67NR cancer cell lines were kindly gifted by Fred Miller (Wayne State University) and cultured in Dulbecco’s modified Eagle medium glutaMAX (DMEM GlutaMAX; Gibco, Thermo Fisher Scientific, cat. no. 10566016, Grand Island, NY, USA) supplemented with 1% penicillin-streptomycin (100 U/mL, Gibco, Thermo Fisher Scientific) and 10% foetal bovine serum (FBS, Gibco, Thermo Fisher Scientific). The 4T1-H2B-GFP+ cells were previously generated by stable transfection of 4T1 cells with a pBOS-H2BGFP vector (BD Pharmingen, San Jose, CA, USA) and were cultured in the same conditions as the 4T1 cells.

BALB/c primary lung (cat. no. BALB-5013), lymph (cat. no. BALB-5070) and mammary (cat. no. BALB-5072) fibroblasts (isolated from healthy mice) were purchased from Cell Biologics (Chicago, IL, USA) and were cultured in Complete Fibroblast Medium with supplement kit (cat. no. M2267, Cell Biologics), which includes 0.5 mL FGF (Fibroblast Growth Factor), 0.5 mL Hydrocortisone, 5 mL L-Glutamine, 5 mL Antibiotic Solution and 50 mL FBS. Primary fibroblasts were cultured on 0.2% gelatin (Sigma-Aldrich, Saint Louis, MO, USA, Gelatin solution, Type B, 2% in H_2_O, G1393-100 mL) coated cell culture dishes. Mouse cancer-associated mammary fibroblasts, mCAF1, were a kind gift from Erik Sahai (The Francis Crick Institute, London, UK). They were isolated from a mammary fat pad of a tumour bearing FVB/n MMTV-PyMT mouse line and immortalised. mCAF1s were cultured in DMEM high glucose (Gibco, Thermo Fisher Scientific) with 10% FBS, 1% Insulin Transferrin Selenium Solution (ITS-G; Gibco, Thermo Fisher Scientific) and 1% penicillin-streptomycin (100 U/mL, Gibco, Thermo Fisher Scientific). All cell lines were maintained at 37 °C, in a humidified atmosphere with 5% CO_2_ and were routinely tested for mycoplasma.

### 2.2. Generating Fibroblast-Derived Matrices

Fibroblast-derived matrices (FDMs) were generated as previously described [13]. For the primary lung, lymph, and mammary BALB/c fibroblasts, the protocol was adjusted to allow deposition of ECM (extracellular matrix) for 21 days, with media change (+ ascorbic acid) every second day. Additionally, during the process of removing the fibroblasts from the deposited ECM, after the addition of extraction buffer (NH4OH-Triton-X-100), FDMs were washed twice in PBS (gently) and 0.5% sodium deoxycholate (Sigma-Aldrich, D6750-500 mg) was added for 60 min at room temperature. Sodium deoxycholate was then removed, the FDMs were washed in PBS and then DNase I was added as previously described. mCAF1 fibroblasts were cultured for 7 days to allow the deposition of ECM, as previously described, and denuded in the same way as the primary fibroblasts, with the additional lysing step.

### 2.3. Immunofluorescence Staining and Imaging Analysis

FDMs for the lung, mammary and lymph nodes were generated in Corning^®^ (Kennebunk, ME, USA) high content imaging 96-well imaging plates (cat. no. CLS4580-10). To image the FDMs before the fibroblast lysing, after ECM deposition, cells were fixed in 4% Paraformaldehyde Solution (PFA), 4% in PBS (Affymetrix, cat. no. 19943 Santa Clara, CA, USA) for 10 min at room temperature in a laminar flow hood. PFA was then removed, and samples washed in PBS. Cells were permeabilised in 0.2% Triton-X-100 in PBS (Sigma-Aldrich, cat. no. T8787-50 mL) for 2–5 min. Cells were blocked in 3% BSA (Bovine Serum Albumin, Sigma- Aldrich cat. no. A8806-5G) for 60 min at room temperature. Primary antibodies were prepared in 1% BSA. Blocking BSA was removed, and cells washed in PBS, after which primary antibodies were added and incubated overnight at 4 °C. After 16 h primary antibody was removed, and cells were washed 3 times PBS-T (0.05% TWEEN^®^ 20 (Croda Internation PLC, UK) in PBS) (TWEEN^®^ 20, Merck, cat. no. P9416-100ML, Darmstadt, Germany). Secondary antibodies were made up in 1% BSA and incubated with the samples for 4–6 h at room temperature (kept dark), after which they were washed 3 times in PBS-T. Samples were then placed in PBS at 4 °C before imaging.

Immunofluorescence imaging was performed on the inverted confocal microscope (Leica SP8, Wetzlar, Germany) with a WLL and HC PL APO CS2 20×/0.75 IMM oil objective. Images were acquired at 1024 × 1024 resolution.

Nidogen 1 antibody was used at a concentration of 1:500 (Rabbit Polyclonal-Acris-cat. no. AP02274SU, Origene, Rockville, MD, USA). Phalloidin (Phalloidin AlexaFluor 633, cat. no. A22287, Invitrogen, Thermofisher Fisher Scientific) was used to visualise actin and was used at a concentration of 1:500. The secondary antibody Alexa 488 (cat. no. A11001, Invitrogen, Thermo Fisher Scientific) was used at a concentration of 1:400. DAPI (Thermo Fisher Scientific, cat. no. 62248) was used at a concentration of 1:1000.

### 2.4. Imaging of Drug Screen

All the following steps were performed on a Microlab Star liquid handling station (Hamilton, Bonaduz, Switzerland). Drug screen plates were fixed on day 3 after drug library added. Plates were fixed in 100 μL/well 10% formalin (Formalin solution 10% neutral buffered, Sigma-Aldrich, cat. no. HT501128-4L) for 10 min. Cells were permeabilised in 0.2% Triton-X-100 in PBS (Sigma-Aldrich, cat. no. T8787-50 mL) for 2–5 min. Next 2× PBS washes are performed, after which DAPI was added at 1 μg/mL for 90 min at room temperature. Plates were then washed ×3 in PBS and placed in 100 μL PBS and stored at 4 °C (dark) until imaging. Imaging was performed on the INCell Analyzer 2200 (GE Healthcare Life Sciences). Images were analysed using the INCell Analyzer Worktstation 1000 software (GE Healthcare Life Sciences). Nuclei were segmented based on the DAPI staining using the Tophat segmentation method. The mean intensity of GFP and DAPI in each nucleus was measured, and the number of GFP positive and DAPI positive cells were counted and compared to controls.

### 2.5. Mass Spectrometry of Fibroblast-Derived Matrices

FDMs were generated in triplicate from the primary lung, lymph, and mammary fibroblasts, following which the fibroblasts were lysed to leave behind the ECM. FDMs were washed in PBS, scraped, and collected. FDMs were then centrifuged at 8000× *g* for 10 min. PBS was then removed, and samples were resuspended in 20 μL GdCl lysis buffer (6M Guanidinium Hydrochloride, 10 mM TCEP, 40 mM CAA, 100 mM Tris, pH 8.5). Samples were then vortexed and boiled at 95 °C for 5 min, then sonicated using the Bioruptor Next Gen (Diagenode SA, Seraing, Belgium) on maximum energy setting (5 × 30 s on/30 s off). Samples were then centrifuged for 1 min at 13,000 rpm and snap-frozen in liquid nitrogen. Sample preparation and mass spectrometry were then performed as previously described [30]. Briefly, after determining protein concentration with Bradford (Sigma-Aldrich, cat. No. 23236), 5 μg was taken forward for digestion. Samples were diluted 1:3 with 10% Acetonitrile, 50 mM HEPES pH 8.5, LysC (MS grade, cat. no. 125-05061 Wako, Osaka, Japan) added in a 1:50 (enzyme to protein) ratio, and samples were incubated at 37 °C for 4 h. Samples were further diluted to 1:10 with 10% Acetonitrile, 50 mM HEPES pH 8.5; trypsin (MS grade, Promega, Madison, WN USA) was added in a 1:100 (enzyme to protein) ratio, and samples were incubated overnight at 37 °C. Enzyme activity was quenched by adding 2% trifluoroacetic acid (TFA) to a final concentration of 1%. Prior to mass spectrometry analysis, the peptides were desalted on in-house-packed C18 Stagetips [31]. For each sample, 2 discs of C18 material (3M Empore) were packed in a 200 μL tip, and the C18 material was activated with 40 μL of 100% Methanol (HPLC grade, Sigma-Aldrich), then 40 μL of 80% Acetonitrile, 0.1% formic acid. The tips were subsequently equilibrated 2× with 40 μL of 1% TFA, 3% Acetonitrile, after which the samples were loaded using centrifugation at 4000× rpm. After washing the tips twice with 100 μL of 0.1% formic acid, the peptides were eluted into clean 500 μL Eppendorf tubes using 40% Acetonitrile and 0.1% formic acid. The eluted peptides were concentrated in an Eppendorf Speedvac, and re-constituted in 1% TFA, 2% Acetonitrile for Mass Spectrometry (MS) analysis.

#### 2.5.1. Mass Spectrometry Data Acquisition

For each sample, peptides were loaded onto a 2 cm C18 trap column (Thermo Fisher Scientific, cat. no. 164705), connected in-line to a 50 cm C18 reverse-phase analytical column (Thermo Fisher Scientific, EasySpray cat. no. ES803) using 100% Buffer A (0.1% formic acid in water) at 750 bar, using the Thermo EasyLC 1200 HPLC system, and the column oven operating at 45 °C. Peptides were eluted over a 140 min gradient ranging from 6 to 60% of 80% acetonitrile, 0.1% formic acid at 250 nL/min, and the Orbitrap Fusion instrument (Thermo Fisher Scientific) was run in a DD-MS2 top speed method. Full MS spectra were collected at a resolution of 120,000, with an AGC target of 4 × 10^5^ or maximum injection time of 50 ms and a scan range of 400–1500 *m*/*z*. The MS2 spectra were obtained in the ion trap operating at rapid speed, with an AGC target value of 1 × 10^4^ or maximum injection time of 35 ms, a normalised HCD collision energy of 30, and an intensity threshold of 1.7 × 10^4^. Dynamic exclusion was set to 60 s, and ions with a charge state <2, >7 or unknown were excluded. MS_2_ performance was verified for consistency by running complex cell lysate quality control standards, and chromatography was monitored to check for reproducibility.

#### 2.5.2. Data Processing

Raw MS/MS spectra were processed using MaxQuant version 1.6.1.0 (Max-Planck-Institut, Germany) and searched against the *mus musculus* reference proteome obtained from Uniprot. Label-free quantitation (LFQ) was enabled, dynamic modifications were set as Oxidation (M) and Acety. on protein N-termini. Cysteine carbamidomethyl was set as a static modification. All results were filtered to a 1% FDR (False Discovery Rate), and common contaminants removed, resulting in a final protein matrix of 3913 proteins identified. For downstream processing, protein identifications with N = 3 in at least one group were retained, and missing values imputed using the built-in imputation algorithm in Perseus (v. 1.5.0.9). The MS proteomics data have been deposited to the ProteomeXchange Consortium (Cambridge, UK) via the PRIDE partner repository with the dataset identifier PXD025786.

#### 2.5.3. ISDoT Mass Spectrometry Acquisition and Data Processing

ISDoT (In Situ Decellularisation of Tissues) and FDM mass spectrometry sample preparation and analysis were performed the same way as described, with the following notable differences [32]. Organs from 8-week-old Balb/c female mice were used (three per condition).

“Following ISDoT decellularisation, organs were thoroughly washed and dissected free of extraneous tissue before being ground in liquid nitrogen to a fine powder, using a chilled mortar and pestle. Solubilised proteins were reduced in 5 mM DTT (Sigma-Aldrich) and alkylated with 10 mM chloroacetamide (Sigma-Aldrich) before deglycolsylation with PNGase F overnight at 37 °C with shaking (100 U per 10 mg tissue).

The Q-Exactive (Thermo Fisher Scientific) was run using the DD-MS2 top10 method. Full MS spectra were collected at a resolution of 70,000, with an Automatic Gain Control (AGC) target of 3 × 10^6^ or a maximum injection time of 20 ms and a scan range of 300–1750 *m*/*z*. The MS_2_ spectra were obtained at a resolution of 17,500, with an AGC target value of 1 × 10^6^ or a maximum injection time of 60 ms, a normalised collision energy of 25%, and an underfill ratio of 0.1%. Dynamic exclusion was set to 45 s, and ions with a charge state of <2 or unknown were excluded.

Raw MS/MS spectra were processed using MaxQuant version 1.5.1.2 software (Max- Planck Institute, Germany) and searched against the mouse protein database UP000000589_10090 (released 25/02/16). Functional-enrichment analysis was performed using High-Throughput GoMiner software57. One thousand randomisations were performed, and the data were thresholded for a 5% false-discovery rate. Over-represented biological-process and cellular-component terms with ≥5 and ≤500 assigned proteins were reported. The fold change in diseased relative to healthy tissue was mapped onto proteins assigned to each over-represented term”.

### 2.6. Kinase Profiling and Analysis

Mammary, lung, lymph node, and mCAF1 FDMs were generated in duplicate and denuded as previously described in 35 mm plastic dishes. Dishes were also coated with Matrigel (Cultrex 3-D culture matrix cat. no. 3445-005-01, lot no. 1519214, R&D Systems, MN, USA), which was diluted in serum-free media (1:1000) to obtain 15 μg/mL overnight at 4 °C. Plates were also coated with 0.2% gelatin (Sigma-Aldrich, Gelatin solution, Type B, 2% in H_2_O, G1393-100 mL) and 0.2% gelatin followed by Matrigel for 4 h. 4T1 cancer cells were then seeded in serum-free media, the same as used for the 4T1 cells (200,000 cells/35 mm well). After 16 h, 4T1 cells on the matrices were lysed and collected on ice in 100 μL Mammalian Protein Extraction Reagent (of M-PER) (ThermoFisher) with ×1 EDTA-free protease and ×1 phosphatase inhibitor cocktails (Halt, ThermoFisher). Samples were then centrifuged (10 min, at 4 °C, 16,000× *g*) and supernatant collected. Protein concentration was determined by Bradford Assay.

A unit of 5 μg of protein was mixed with FITC-conjugated anti-phophotyrosine antibody PY20 from each sample and was loaded onto either the PTK (protein tyrosine kinases) or STK (serine/threonine kinases) PamChips (Pamgene, Hertogenbosch, The Netherlands, which contain porous membrane that contains peptides with known phosphorylation sites. When loaded onto the Pamstation12 (Hertogenbosch, The Netherlands), the samples were then pumped through the membrane of the chips, and which allowed peptides on the membrane to be phosphorylated. Fluorescence emitted was then imaged by a built-in CCD camera [33,34].

Raw images were then analysed using BioNavigator software (Pamgene, Hertogenbosch, The Netherlands). *T*-test of the relative signal intensities of peptides changed was performed (Table S5, performed in GraphPad Prism v. 9.02, (San Diego, CA, USA). Predicted upstream kinases were analysed using the Upstream Kinase Analysis App (v.2018, BioNavigator, Pamgene), where kinase activity is based on multiple upstream kinase databases as is reported as a mean kinase statistic. Hierarchical clustering of the kinase activity across the samples was performed in Cluster 3.0 (C Clustering Library 1.59 for Mac OS X, Tokyo, Japan) and visualised using Java Tree View (v.1.2.0, Boston, MA, USA).

### 2.7. Kinase Inhibitor Drug Screen

Kinase inhibitor library (Inhibitor Select^TM^ Protein Kinase Inhibitor Library I (cat. no. 539744, Calbiochem Germany), Library II (cat. no. 539745), and Library III (cat. no. 539746) used were kindly gifted by Professor Michael Givskov. Inhibitors were at a 1 mM stock solution in dimethyl sulfoxide(DMSO). Working stock solutions were prepared as follows: dilute to 100 μM in 10% DMSO and 90% PBS. For each library, 10 μL of drug was then aliquoted into 1 ‘working’ drug plate per repeat. At time of use, 90 μL of 2% serum media was added to each drug to make a 10 μM solution in 1% DMSO, 9% PBS, and 90% media. A unit of 20 μL of each drug at 10 μM was then added to 180 μL of media and cells (2% FBS) with a final drug concentration of 1 μM.

#### 2.7.1. Primary Lung Fibroblast and mCAF1 Kinase Inhibitor Drug Screen

Primary lung FDM and mCAF1 FDM was prepared as previously described in Corning^®^ high-content-imaging 96-well imaging plates (cat. no. CLS4580-10). 15,000 cells per well were seeded. After the fibroblasts were denuded, the lung FDMs were placed in PBS while the 4T1-H2B-GFP cells were prepared. A total of 3000 4T1-H2B-GFP cells were added to each well in 180 μL 2% FBS complete media, and after 16 h the kinase inhibitor drug libraries were added. After 3 days, plates were fixed and imaged. Each plate of the drug screen within each repeat had a DMSO control (in triplicate) present. In addition, edge wells were not used for screening to limit edge effects and were filled with 200 μL PBS.

#### 2.7.2. Matrigel and Gelatin Kinase Inhibitor Drug Screen

Corning^®^ high-content-imaging 96-well imaging plates (cat. no. CLS4580-10) were coated with Matrigel overnight (Cultrex 3-D culture matrix cat. no. 3445-005-01, lot no. 1519214,), which was diluted in serum-free media (1:1000) to obtain a 15 μg/mL solution at 4 °C. Plates were also coated in 0.2% gelatin (Sigma-Aldrich, Gelatin solution, Type B, 2% in H_2_O, G1393-100 mL) overnight at 4 °C. Before use, coatings were washed in PBS and then 3000 4T1-H2B-GFP cells were seeded, and kinase inhibitor library added as above.

#### 2.7.3. Cell Viability

To perform cell viability assays, 4T1 cells were added to FDMs or plastic in 2% FBS medium. RealTime-Glo (Promega, cat. no. G9712) was then added at ×5 concentration (stock ×1000) and incubated for 60 min at 37 °C, after which luminescence (578 nm) was read using the SpectraMax Paradigm multi-mode detection platform. Luminescence reading was then taken every 24 h for a total of 72 h.

### 2.8. Statistical Analysis

All statistical analyses were performed in GraphPad Prism 9 (GraphPad Software, San Diego, CA, USA) unless otherwise stated. Data were tested for normal distribution, following which the appropriate statistical analysis was chosen. Drug inhibition was tested using a 2-way ANOVA with multiple comparisons (corrected using Šídák’s hypothesis testing). Adjusted *p*-values were reported with a confidence level of 0.05 (95% confidence interval). Simple linear regression analysis was performed to find the best-fit value of the slope and intercept.

### 2.9. Generating Protein Interaction Networks

ECM proteins that were identified in lung FDM, kinases that were activated by lung FDM, and targets of drugs that reduced 4T1 proliferation on lung FDM were assembled. Interaction networks were generated using Cytoscape (version 3.8.2, San Diego, CA, USA) [35] and Genemania (version 3.5.2, Toronto, Canada) [36] as previously described [37]. Lines between nodes (proteins) represent reported physical protein–protein interactions, and up to 100 additional interacting proteins were automatically added to assist with hypothesis generation. Finally, proteins with no interacting partners were removed and the yFiles organic layout [38] was applied.

## 3. Results

### 3.1. Primary and Metastatic Site Fibroblasts Produce Complex ECM

**Figure 1:**
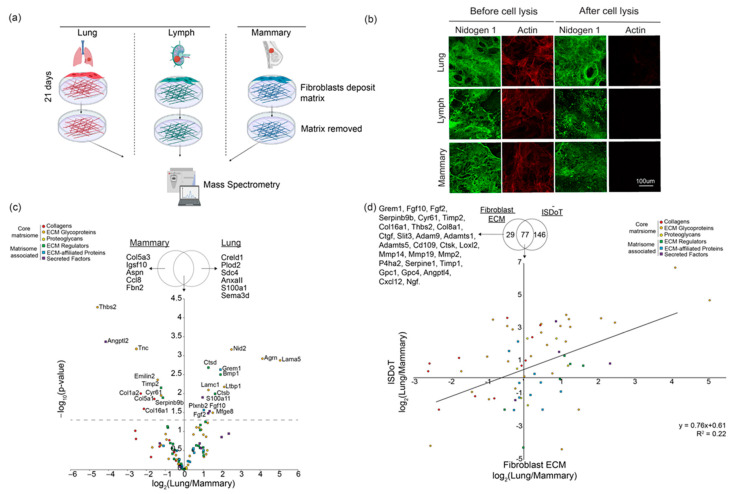
(**a**) Schematic showing the process of fibroblast-derived matrices and overview of experimental design. (**b**) Immunofluorescence imaging illustrating the lung, lymph, and mammary fibroblast matrices and deposited matrix, which is left intact post-lysis (visualised with Nidogen 1, green). Fibroblasts stained for actin (red). (**c**) Results of quantitative mass spectrometry, filtered for matrisome, of lung vs. mammary fibroblast-derived matrix (–log_10_ *p*-value 1.3 indicates proteins significantly higher in each condition). Venn diagram displays which lung and mammary ECM proteins were detected in those conditions. (**d**) Comparison of ECM proteins between lung and mammary fibroblast-derived matrix, and lung and mammary decellularised organs using the ISDoT method. Simple linear regression of data shows R2 value of 0.22. Venn diagram shows that 77 ECM proteins were detected in both data sets, 29 ECM proteins were only detected in the FDMs, and 146 proteins in decellularised organs.

Primary murine BALB/c fibroblasts from the lymph nodes, lung, and mammary fat pad were cultured for 21 days in order to allow the deposition of FDM (fibroblast derived matrix) using the previously established method [13] (Figure 1a). After 3 weeks of ECM (extracellular matrix) deposition, fibroblasts are successfully denuded (actin staining, Figure 1b), whereas the ECM is retained as shown by the continued presence of the ECM component nidogen 1 after fibroblast lysis (Figure 1b, Figure S1a,b).

To determine differences in ECM composition between organs, FDM from the lymph node, lung, and mammary fat pad was analysed using label-free quantitative mass spectrometry, which allows quantification of the relative abundance of proteins between conditions. To ensure that the fibroblasts (which are isolated from healthy mice) did not become activated, cells were cultured on gelatin-coated plates. Detected proteins were filtered using the in silico ‘matrisome’ generating a list of known ECM proteins (106 proteins, Table S1) detected in the lymph node, lung, and mammary FDMs. ECM proteins in the different conditions were grouped according to function and included collagens, ECM glycoproteins, proteoglycans, ECM regulators, ECM-affiliated proteins, and secreted factors, showing that we were able to detect a complex mixture of ECM proteins in the lymph, lung, and mammary FDMs.

When comparing lung and mammary FDM, 14 proteins were significantly higher in abundance (*p*-value < 0.05) in the lung FDM, and 10 were significantly higher in mammary (Figure 1c). We also saw that a subset of ECM proteins was only detected in one of the organs, such as Col5a3, Igsf10, Aspn, Fbn2 and Ccl8, that were only seen in the mammary FDM. In the lung, only Creld1, Plod2, Sdc4, Anxa11, Sema3d, and S100a1 were specifically detected. Comparison between the lung and lymph node FDMs revealed 46 ECM proteins to be significantly more abundant in the lung FDM (*p*-value < 0.05), while in the lymph node, only 11 ECM proteins were significantly more abundant (*p*-value < 0.05) (Figure S1a). Furthermore, when comparing the primary site (mammary) and lymph, the mammary fibroblasts produced 29 ECM proteins that were significantly higher (*p*-value < 0.05) than the lymph node, while only 6 ECM proteins were lymph-enriched (Figure S1b). These results indicate that the ECM composition produced from different organ fibroblasts differ from each other and are composed of a complex mixture of ECM proteins. Additionally, using our previously published proteomics data from the ISDoT method (In Situ Decellularisation of Tissues) [32] (Table S2), we compared the composition of the lung vs. mammary FDMs with in vivo decellularised lung vs. mammary ECM (Table S3). Of the 223 proteins identified from in vivo organs, 77 were detected in FDMs. The proteins not detected in FDMs are likely produced by other cell types. Conversely, 29 ECM proteins were identified in FDMs but not from in vivo organs, which are likely small and/or low abundance proteins that are hard to detect in vivo and include growth factors and cytokines. When comparing the quantitative changes between lung vs. mammary FDMs and ISDoT, we see that for 55 proteins, their abundance either increases or decreases in both FDMs and in vivo (Figure 1d).

### 3.2. Primary and Metastatic Site ECM Induce Differential Kinase Signalling Profiles in 4T1 and 67NR Breast Cancer Cells

**Figure 2:**
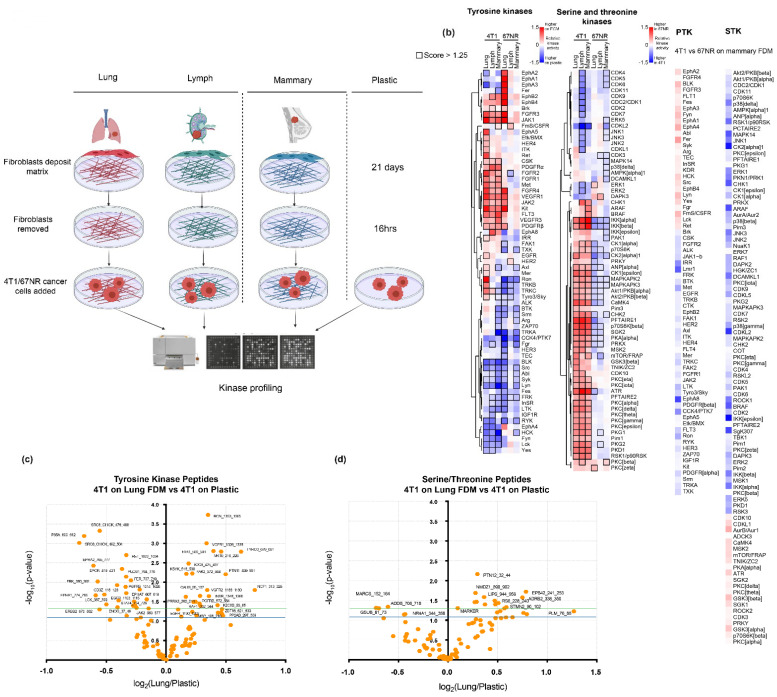
(**a**) Schematic representation of kinase signalling of 4T1/67NR experimental design where FDM is derived from the lung, lymph, and mammary fibroblasts. Fibroblasts are then denuded, and cancer cells seeded on the FDMs and plastic, after which lysates are collected, and kinase profiling is performed. (**b**) Heatmaps comparing Mean Kinase Statistic (relative kinase activity) across the following conditions, 4T1/67NR lung vs. plastic, lymph vs. plastic, and mammary vs. plastic for both tyrosine kinases and serine/threonine kinases (red = higher on FDM). Kinases are clustered hierarchically. Kinase values with a score above 1.25 are enclosed in a black border, representing changes in kinase activity that has reached a reliable threshold. Additionally, kinase activity of 4T1s vs. 67NR on mammary FDM where red kinases show those activated in 67NRs. (**c**,**d**) Volcano plots show the levels of phosphopeptides detected in 4T1 cells between lung and plastic. Peptides that reached statistical significance (*p*-value > –log_10_ 1.3) are indicated by the green line.

FDM ECM analysis revealed altered ECM composition between different organs; consequently, we sought to determine whether these ECM organs induce different signalling in cancer cells, which could help to identify signalling pathways involved in cancer cell growth, specifically at metastatic sites. 4T1 cells were therefore seeded onto intact FDMs from the lymph, lung, and mammary, and, as a control, plastic, and were allowed to attach and proliferate for 16 h in serum-free media (Figure 2a). We used both 4T1 (invasive) and 67NR (non-invasive) breast cancer cells to enable us to identify the most promising signalling pathways for metastasis. Cancer cell lysates were collected for each condition and were analysed for global tyrosine kinase (PTK) and serine/threonine kinase (STK) signalling using the Pamgene kinase profiling system. The kinome of each sample was analysed using PTK/STK chips, the membrane of which contains a microarray of peptide sequences (196 PTK peptides, 144 STK peptides). Each peptide incorporates a phosphorylation site which can be correlated with one or multiple upstream kinases responsible for its phosphorylation. After addition of the cancer cell lysate and ATP, active kinases phosphorylate peptides, the relative levels of which are quantified using fluorescence intensity [39]. Based on known kinase–substrate relationships, predicted upstream kinase activity between different conditions can also be determined.

Here, we obtain an overview of global kinase signalling of both 4T1 and 67NR breast cancer cell lines, when they are on different organ FDMs, and importantly obtained an overview of the differences between primary and metastatic site FDMs. First, we analysed the changes in phosphorylation at the peptide level. We observed that 19 peptides had increased phosphorylation across both cell lines compared to plastic (Figure S2, Table S6). When comparing between celllines, we see that the patterns of phosphorylation of the peptides separate according to cell line, with 11 PTK peptides and 5 STK peptides showing an increase in phosphorylation in the 4T1′s on plastic vs. ECM, but not in the 67 NRs (Figure 2c,d).

Using known kinase–substrate interactions, we were then able to correlate the changes in phosphorylation at the peptide level with changes in kinase activity of the cells when they are on the FDMs vs. plastic. Here, we wanted to elucidate kinase activation patterns or pathways that could then be targeted in breast cancer metastasis.

Here we observe several kinases which are activated on FDMs vs. plastic in both the PTK and STK (Figure 2b). We show that the kinase signalling patterns clearly separate by cell line, with differences seen in kinase activation between the 4T1s and 67NRs. In the 4T1s, compared to plastic, we see that ITK, IRR, PKCα/δ/γ/φ/ε, PKG1/2, Pim1, PKD1, RSK1, IKKα/β, ANP, CK1, MAPKAPK2/3, Akt1/2, CamK4, p7056kβ, SGK, PDGFRα/β, FGFR, pKA, and PRKX are activated in the 4T1s but not in the 67NRs (across all FDM types compared to plastic). These are of interest, as they may be specific to the invasive capabilities of the 4T1 cells. Furthermore, we observe that a number of kinases previously reported to be involved in breast cancer such as PDGFRα, PDGFRβ (platelet-derived growth factor receptor α/β) [40], FGFR (fibroblast growth factor receptor) 1/2/3/4 [41], Akt 1 and Akt2 [42], and Pim1 [43] are also activated, validating our data and strengthening the reliability of our results. Furthermore, many of these kinases interact with each other via other known kinases such as EGFR (epidermal growth factor receptor) or are within the same pathways, such as Akt1 and MAPKAPK2, which are both part of the p38 MAPKinase pathway [42], which further expands which further expands the possibilities of targeting the metastatic development of 4T1 cells.We also see that when we compare the kinase activation of 67NRs and 4T1s at the primary site (mammary), a number of kinases are activated in the 4T1s alone, which could indicate kinases that are involved in the invasiveness of 4T1 cells. We also see more kinases activated in the 67NRs on the mammary FDM, which shows nicely that these non-invasive cells are more activated at the primary site, where it is expected that they promote primary tumour growth.

We then investigated kinase changes induced by the specific FDMs, looking at kinases activated between the metastatic (lymph node and lung) and primary site (mammary). When we compared the lung FDM with mammary FDM, we see that there are a small number of tyrosine and serine/threonine kinases that are activated on the lung, but not on the mammary ECM such as EphA5, Tyro3, Ron, TRKB, CK2alpha, and Pim3 (Figure 2b). This is important, as subtle changes in the signalling of the cancer cells specific to the lung FDM, may elucidate pathways that could be involved in cancer progression at that site specifically. However, we see that the signalling changes in response to FDMs of different composition are minor compared to the distinctive changes in activated kinase signalling pathways in 4T1 breast cancer cells and 67NRs depending on whether they are on ECM or plastic.

### 3.3. Small Molecule Kinase Inhibitors Show Different Efficacy When Cancer Cells Are Cultured on ECM or Plastic

We have shown that signalling patterns of cancer cells on different ECM organs are similar but are different when culturing cancer cells on plastic. Therefore, we next sought to investigate whether ECM- vs. plastic-induced signalling could lead to altered drug responses when assessing cancer cell growth.

**Figure 3:**
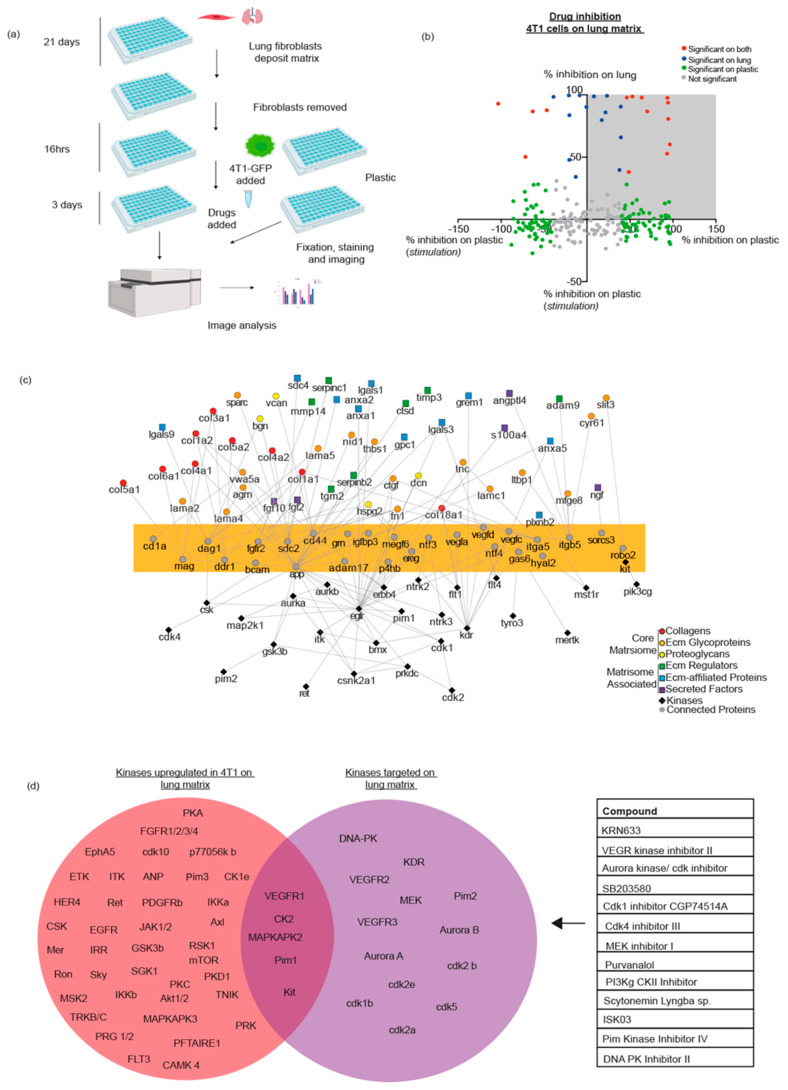
(**a**) Schematic showing the experimental workflow used for the kinase inhibitor drug screening on the lung FDM. Fibroblasts are treated to deposit ECM for 21 days, after which they are removed and 4T1-H2B-GFP cells are seeded. After 16 h, the drug library is added, and after 3 days, cells are fixed, stained, imaged, and analysed. (**b**) X/Y plot showing drugs that only inhibited cell growth on the lung matrix, plastic or on both. Colours represent statistical significance in inhibition (*p*-value < 0.05) of 4T1 cells either on the lung FDM, plastic, or both substrates. (**c**) Network analysis linking the ECM proteins, which are enriched in the lung FDM with kinases which are up-regulated in 4T1s on the lung FDM vs. plastic. Physical protein–protein interactions are linked (grey circles) and proteins highlighted in yellow are receptors and proteins predicted to link the ECM and kinases. (**d**) Venn diagram; in the red sphere showing a list of kinases which are up-regulated in 4T1 cells on the lung FDM (both PTK and STK). Purple sphere contains the kinases which were successfully targeted on the lung matrix when the kinase inhibitor drug screen was performed. Overlap represents kinases that crossover. Table showing the drugs which significantly inhibited 4T1 cancer cell proliferation, specifically on the lung FDM.

We used a library of 242 small molecule kinase inhibitors (a mixture of both STK and PTK inhibitors—see Table S4) on 4T1 cells cultured on either FDM or plastic. Briefly, we generated FDMs from the primary lung fibroblasts, denuded after 21 days and plated GFP labelled 4T1 cells (Figure 3a). The drug library was used at a concentration of 1 uM [44,45] and was added after 16 h to modulate the pathways that were measured in our kinase data. This concentration was chosen, as we saw a reduction in proliferation with our positive control Dasatinib (MedchemExpress, USA), which is a known kinase inhibitor of proliferation [46]). After 3 days (the optimal time for cells to proliferate in 2% serum media), plates were fixed and imaged, and the number of GFP positive cells were counted. We compared the proliferation of 4T1 cells treated with a drug compound to vehicle control (DMSO), and also included untreated and Dasatinib-treated cells, as further controls. Interestingly, we show that 4T1 cells proliferate at a higher level on plastic vs. lung FDM (Figure S3a). We compared the proliferation of 4T1 cells treated with a drug compound to vehicle control (DMSO). Additionally, we included untreated cells, and Dasatinib-treated, as a further control. We saw that 10 drugs significantly inhibited 4T1 growth on both plastic and lung ECM, while 21 drugs inhibited proliferation only when cells were on plastic (Figure 3b, *p*-value < 0.05) (Table S5). Furthermore, 13 drugs significantly inhibited the proliferation of 4T1 cells on only the lung FDM and not on plastic (Figure 3b) (*p*-value < 0.05). Moreover, when comparing to the kinase signalling of 4T1s on lung FDM, we can see that the signaling pathway of several of these targets were also activated on ECM compared to plastic in the 4T1 cells, such as CK2, VEGFR1, Kit, MAPKAP2, and Pim1 (Figure 3d). A subset (CK1/2 inhibitor, VEGFR inhibitor, and Akt inhibitor) of drugs which significantly reduced 4T1 proliferation on the lung FDM were then further tested at different doses and viability was measured using RealTime-glo. We see that these inhibitors reduce the viability of these cells in a dose-dependent manner (Figure S3b,c).

Here, we show the feasibility of using complex FDMs for high-throughput drug screening. We demonstrate that the use of plastic and FDM for screening of kinase inhibitors yields dissimilar results, with selected drugs only inhibiting 4T1 cell proliferation when on FDM, some on plastic, and some on both culture conditions. These data suggest pathways that may be more relevant in the context of breast cancer cell growth in the lungs, as they specifically inhibit cancer cell growth on lung FDM.

Importantly, we show here that using the lung FDM for drug testing, compared to plastic, changes the potential targets that could be further investigated, as we find drugs that inhibit only on the lung ECM and would therefore have been overlooked if drug screening was performed on plastic alone. We also observe that some inhibitors are only efficacious on plastic but not on the lung FDM (which we hypothesise has more in vivo relevance in metastatic breast cancer), which could also help in reducing false positives and identifying pathways that are of most clinical importance.

### 3.4. Generating Further Hypothesis Using Proteomics and Kinome Data

We then investigated how our kinase data, drug screen analysis, and proteomics data relate to each other, and which other proteins may be relevant to breast cancer metastasis to the lung. We therefore generated a protein–protein interaction network model by looking at the ECM-enriched proteins in the generated lung FDM and the kinases that are activated in 4T1 breast cancer cells, when they interact with the lung FDM. We observe several protein interactions and highlight several receptor proteins (yellow highlight, Figure 3c) that could mediate interactions between ECM components and upstream kinases. These include glycoproteins such as CD1A, MAG, DAG1, BCAM, and CD44, integrins such as ITGA5 and ITGB5, and other well-known receptors and proteins such as FGFR2, DDR1, APP, VEGFA/D/C, and ADAM17. We see that APP, a cell surface receptor, links kinases such as EGFR (and further downstream Pim1) to multiple ECM proteins. Additionally, we sought to investigate which kinases were only targeted on the lung FDM in the kinase inhibitor drug screen. We find that Pim1, MAPKAPK2, and CK2 are kinases that are activated on the lung FDM, and were inhibited on the lung ECM alone, which would be good candidates to investigate when looking at breast cancer metastasis to the lungs (Figure 3d).

### 3.5. Kinase Signalling and Drug Response of 4T1 Cells on Different ECM Substrates

**Figure 4:**
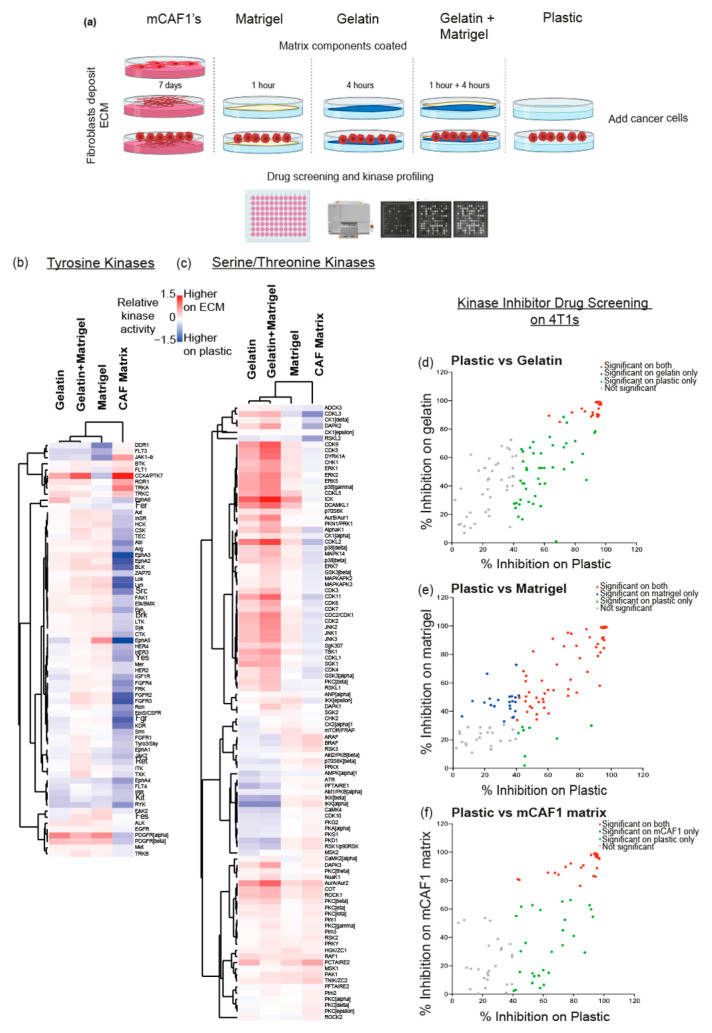
(**a**) Schematic showing the experimental design used for follow-up studies. mCAF1 fibroblasts are seeded and cancer-associated matrix is derived, following which fibroblasts are removed and 4T1 cells are seeded. In addition, 4T1 cells are seeded on Matrigel, gelatin and Matrigel, and gelatin-coated plates, as well as plastic. Drug screening and kinase profiling were then performed. (**b**,**c**) Heatmaps showing the results of the kinase signalling of 4T1 cells on gelatin, Matrigel and gelatin, and Matrigel and mCAF1 matrix for both PTKs and STKs compared to plastic. Kinases indicated as ‘red’ show higher levels of activation. (**d**) Inhibition of 4T1s on gelatin vs. plastic. (**e**) Inhibition of 4T1 cells on Matrigel vs. plastic. (**f**) Inhibition of 4T1 cells on mCAF1 matrix vs. plastic. Significance defined as adjusted *p*-value < 0.05.

Having shown that the FDM produced by different organ fibroblasts induces similar kinase activation in 4T1 cells, but different to when 4T1 cells are on plastic, we sought to compare kinase signalling signatures of 4T1 cells on a broader range of ECMs (Figure 4a). Cancer-associated fibroblasts (CAFs) have been shown both in vitro and in vivo to promote breast cancer tumour progression at the primary site [47]. While the healthy organ fibroblasts used so far can give us a clear picture of inter-organ differences when cancer cells arrive at native ECM, often in a cancer setting, fibroblasts at the primary and metastatic sites have become ‘activated’. This activation leads to changes in ECM and promotes cancer dissemination and progression [6]. Here, we used the mCAF1 cell line derived from the mammary fat pad of tumour-bearing MMTV-PyMT mice [48] in order to recapitulate this. In addition, we repeated signalling studies with two substrates which are often used in vitro to recapitulate the ECM: Matrigel (basement membrane components), gelatin (digested collagens), and gelatin and Matrigel together, as these are simpler, commercially available materials that are easy to obtain and use in the laboratory.

When looking at global kinase signalling, again, we observe that several tyrosine and serine/threonine kinases are activated on each of the different substrates, compared to plastic (Figure 4b,c). Additionally, we verified these at the peptide level (Figure S4, Table S7). Interestingly, we find that the mCAF1 FDM induces a different signalling pattern compared to the other ECMs used. Indeed, it appears that when 4T1 cells are on the cancer-associated FDM, this leads to the de-activation of many kinases which we saw activated on the lung FDM, such as Ron, MAPKAP2, and CK2 (Figure 2b). However, Pim1 kinase, which was previously activated in a lung metastasis context (lung FDM), remains activated on the mCAF1 FDM. This further supports Pim1 as a potential target in breast cancer metastasis. Furthermore, we see that activation patterns of kinases in 4T1s on gelatin and Matrigel + gelatin, are similar to each other as we show (via hierarchical clustering, Figure 4b) that signalling on Matrigel shares characteristics with all the ECM substrates, as well as mCAF1 FDM.

Thus far, we have shown that the drug response of breast cancer cells is changed if the cells are exposed to the ECM derived from lung fibroblasts, compared to plastic. We therefore decided to investigate whether the use of FDMs from CAFs and the other ECM components produced similar results. In addition to using Matrigel and/or gelatin-coated plates, mCAF1 cells were cultured, allowed to deposit ECM and denuded, following which 4T1 cells were seeded and the drug screen was performed. These additional screens revealed that several inhibitors are effective on plastic but not on either the mCAF1 FDM, gelatin, or Matrigel (Figure 4c–f).

Again, we see that the use of an ECM component is important as drug inhibition on plastic alone would have excluded potentially important targets. Comparing the Matrigel drug screen to its kinase activation profile also shows an overlap, with 25 inhibitors reducing the proliferation of 4T1 cells significantly on Matrigel alone. Of these 25 inhibitors, they target 17 kinases seen to be ‘up-regulated’ in 4T1s on Matrigel compared to plastic, which validates the use of Matrigel in this context.

In this study, we have highlighted the importance of including the ECM during drug screening in the pre-clinical setting. Our data clearly show that, even when cancer cells are on FDMs from different organs or on ‘simpler’ ECMs, drug response and kinase signalling patterns differ greatly than when they are on plastic.

## 4. Discussion

Knowing the important role that the ECM plays during metastatic progression of breast cancer, we adapted a cell-based approach to derive in vitro native ECM and investigated kinase signalling and drug response in a model of metastatic breast cancer. We used normal fibroblasts from known metastatic organs in breast cancer (lymph nodes and lungs) as well as the primary site (mammary gland). These FDMs closely resemble in vivo the ECM components of decellularised lungs vs. mammary, and we show by mass spectrometry that the FDMs generated are complex and abundant in ECM constituents.

Cancer–ECM interactions are critical during metastatic progression. In order for cancer cells at the primary tumour site to travel to local and distant organs, they must come in contact with and move through the ECM within and surrounding the primary tumour (invasion), followed by breaching of the basement membrane (intravasation), and then the reverse at the secondary site (extravasation) [49]. At all times, the ECM is providing biophysical support and biochemical cues that affect the ability of cancer cells to proliferate and invade, which could be targeted by therapeutics [50]. The ECM during cancer progression is often altered in terms of stiffness, structure, arrangement, and composition [4] and as such, provides a unique microenvironment in which cancer cells can activate intracellular signalling pathways necessary for proliferation and migration. We also see that our mammary FDMs have significantly increased collagen I levels compared to the lung FDM, which recapitulates the in vivo situation, as our previous proteomics also shows an increase in collagen I. Increasingly, ‘simpler’ ECM components have also been the focus of cancer studies to include a relevant tumour microenvironment. However, they are often limited in their ability to recapitulate these cancer niche properties as products such as Matrigel and gelatin are only composed of a limited number of ECM components and lack resemblance to native tissue architecture and biophysical properties [51]. The presence of the ECM is also critical when studying cancer cell behaviour in vitro to aid in increasing the efficacy of drugs tested in vitro to patients, as current studies suggest fewer than 5% of all drugs tested make it to the clinic [52,53]. To our knowledge, using FDMs to investigate global kinase signalling and for use as a high throughput drug screening platform has not been previously performed.

We show that irrespective of which organ-specific FDM the 4T1 breast cancer cells are in contact with, overall, there are only minor changes in kinase activation and de-activation. Cancer cells bind to the ECM through integrins and glycoproteins, which transduce signals from the tumour microenvironment into the cell. Integrins are versatile transmembrane proteins that are able to bind to different ECM components such as collagen, laminins, and fibronectin [54]. We therefore posit that if there are a range of different ECM components present surrounding the cancer cells, integrin-mediated kinase signalling can be induced. Further in-depth investigations looking at which specific ECM components (or redundancy in the system) induce key signalling pathways, and through which receptors signalling occurs, will be very beneficial to better understand the ECM-cancer cell interaction in breast cancer. While not directly studied here, the role of specific, important signalling pathways, such as those related to TGFβ, are also likely to be altered and contribute to the results we see. TGFβ has been shown to activate fibroblasts within the tumour microenvironment and therefore induce the production of lysyl oxidase, via SMAD, leading to increased collagen crosslinking and metastasis in breast cancer [55]. We see that both in the mammary and lung FDM, lox is significantly increased in lung vs. mammary. Furthermore, it would be beneficial in this system to further explore whether other functions of the cancers cells are altered via looking at migration and invasion. Multiple studies have shown [56] that FDMs can be used to perform migration studies as they produce the necessary collagen structures to allow this [13,57].

The organ-specific fibroblasts that we used in this study were derived from resident fibroblasts that are present within the organ of healthy mice. While this gives us a good opportunity to look at differences in inter-organ composition during tumour progression (when cancer cells arrive at a new organ) and kinase signalling, during breast cancer progression, cancer cells are in contact with CAFs as well as resident fibroblasts. CAFs have been shown to remodel and reorganise the ECM, provide tracks for cell migration, and physically pull cancer cells through the tumour microenvironment, aiding in cancer dissemination [7]. We see that when on the mCAF1 FDM, kinase signalling is different to the other FDMs and observe that numerous kinases were de-activated on mCAF1 FDM compared to plastic. Furthermore, we saw that there were no mCAF1 FDM-specific inhibitors. Our study therefore highlights the need for further investigations including the difference between CAF and normally secreted FDM in order to provide better in vitro drug testing and kinase signalling platforms. We also hypothesise that the ECM deposited by the mCAF1s leads to lowered kinase activation levels across many kinases that are important for cell proliferation (such as cyclin-dependent kinases). When these levels are ‘lowered’, drugs that target kinases which are normally highly activated in cancer cells are no longer as efficacious. It would therefore be of interest to see whether the use of combination treatments of kinases inhibitors and PP2A (Protein Phosphatase 2) activators such as SMAP (small molecule activator of PP2A), that allows the re-activation of kinase signalling pathways [58], could lead to re-sensitisation to certain kinase inhibitors and increased efficacy.

We have shown that 4T1 cells on different ECM types display only subtle changes in cell response, despite differences in ECM composition. However, we observe very large differences in both kinase signalling and, more importantly, drug response when 4T1 cells are seeded on plastic. We show that distinctive kinase signalling pathways are activated in 4T1 breast cancer cells and 67NRs depending on whether they are on ECM or plastic, while comparatively minor signalling changes were observed in response to FDMs of different composition. Kinases that could be used as targets at the metastatic lung site would might be EphA5, Tyro3, Ron, TRKB, CK2alpha, and Pim3; however, these kinases display lower changes in activation in the lung compared to mammary. Kinases that would be of interest, as targeting possibilities for metastasis in general in breast cancer would be those that are activated on the FDMs vs. plastic, and in the 4T1, such as ITK, IRR, PKCα/δ/γ/φ/ε, PKG1/2, Pim1, PKD1, RSK1, IKKα/β, ANP, CK1, MAPKAPK2/3, Akt1/2, CamK4, p7056kβ, SGK, PDGFRα/β, FGFR, pKA, and PRKX. These could also be further investigated as biomarkers in breast cancer, as changed expression levels may correlate with metastasis-free survival, particularly to the lungs.

Conventional drug screening platforms performed in vitro are often 2D and use plastic as a substrate. Increasingly, pre-clinical cancer research is moving towards using models which are 3D and encompass the greater complexities of the tumour microenvironment to better model diseases and test drugs. However, progress in this area is hindered as platforms such as these are difficult to ‘scale-up’. Matrigel-based approaches to drug screening are common in organoid cultures, where primary cells from patient tumours are embedded within 3D Matrigel. While these cultures often maintain the genomic properties of the tumours, and encompass cell heterogeneity, they have can be difficult to maintain [59]. While other Matrigel-based cultures such as MBOC (Matrigel bilayer organoid culture) developed for gynaecological tumours [60] have been shown to improve the viability of cell cultures and allow for drug screening, they yet do not encompass the complex mix of ECM proteins that are deposited by fibroblasts. FDMs, while they provide a relevant tumour microenvironment, are easily manipulated, which limits their versatility. Furthermore, fibroblasts in culture change their phenotype (such as an increase in αSMA expression [61]) to varying degrees, which requires careful phenotypic and molecular monitoring. A good test of our drug screening platform would be to see whether a clinically effective kinase therapy was effective on our FDMs but not on other 2D substrates. This would demonstrate the further clinical relevance of FDMs. Here, we show that we can use FDMs to perform high-throughput drug screening and highlight that using an ECM substrate during this process is of critical importance.

In our drug screens, we treated 4T1 cells with kinase inhibitors at one concentration (1 μM) for a set time (3 days). The inhibitory capabilities of these inhibitors differ when 4T1 cells are on plastic or ECM. There are drugs that show efficacy when 4T1s are on plastic but not on ECM, irrespective of the matrix substrate (gelatin, Matrigel, or lung FDM), which we hypothesise have less clinical relevance, as they do not encompass the complex protein composition seen in vivo. We know the ECM can act as a barrier to drug delivery to cancer cells [62] and, as such, is a vital element in drug testing. The differences that we see in drug response to kinase inhibition are also validated as the global kinase signalling that we have performed clearly shows that activation of kinases differs greatly between 4T1 cells on ECM and plastic.

Here, we present a novel method of performing large-scale drug screening of kinase inhibitor, which encompasses the ECM by using FDMs. While we provide several ECM substrates that could be used to represent the ECM for drug screening, we observe that the FDMs contain the complex mixture of ECM proteins, in addition to other biophysical cues, that might best represent the in vivo situation. We also show that using matrices from different metastatic organs in breast cancer can elucidate subtle changes in kinase signalling pathways that could be targeted. Moreover, in this study, we highlight that there is a real need to incorporate the ECM into in vitro drug screening as results from screening on plastic alone do not replicate those of 4T1 cells on ECM. We therefore stress the importance of including ECM during in vitro breast cancer metastasis investigations, including drug screening.

## 5. Conclusions

This study aims to explore the role of the ECM on breast cancer at different metastatic sites. The conclusions of this study are as follows:

1. ECM produced by fibroblasts from different murine organs (to which breast cancer metastasise) is complex, abundant in ECM constituents, and differ in composition between organs.
2. FDMs can be used to assess global kinase signalling of 4T1 and 67NR breast cancer cells in vitro. Inter-organ kinase activation differs to a smaller degree compared to cancer cells on plastic.
3. FDMs can be used to perform high-throughput screening of kinase inhibitors on 4T1 cells. Inhibition of 4T1 proliferation on ECM differs from 4T1 cells screened on plastic.
4. Network analysis performed proposes targets to inhibit proliferation of 4T1 cells in the lung by combining lung ECM changes with kinase activation profiles, and inferring protein–protein interaction pathways.

## Data Availability

The mass spectrometry proteomics data have been deposited to the ProteomeXchange Consortium via the PRIDE partner repository with the dataset identifier PXD025786.

## Supplementary Materials

The following are available online at https://www.mdpi.com/article/10.3390/cancers13133331/s1, Figure S1: (A) Results of quantitative mass spectrometry, filtered for matrisome, of lymph vs lung fibroblast derived matrix (−log10 *p*-value 1.3 indicates proteins significantly higher in each condition). Venn diagram displays which lung and lymph ECM proteins were only detected in the lung ECM. (B) Results of quantitative mass spectrometry, filtered for matrisome, of mammary vs lymph fibroblast derived matrix (−log10 *p*-value 1.3 indicates proteins significantly higher in each condition). Venn diagram displays which lymph and mammary ECM proteins were only detected in the mammary ECM, Figure S2: Hierarchical clustering of tyrosine and serine/threonine peptides of 4T1 and 67NR cells on lung, lymph and mammary FDMs compared to plastic, Figure S3: (a) Cell viability of 4T1 and 67NR cells on different substrates (lung, mammary and plastic). (b) Cell viability of 4T1 cells (72 hrs) when treated with 3 compounds which had previously reduced 4T1 proliferation on lung FDM (drug screen). (c) Dose response of 4T1 viability for 3 compounds (at 1 µM), Figure S4: Hierarchical clustering of tyrosine and serine/threonine peptides of 4T1 cells on gelatin, gelatin + Matrigel, Matrigel and mCAF1 FDM compared to plastic, Table S1: Proteomics analysis of organ fibroblast-derived matrix, Table S2: Proteomics analysis of lung and mammary ISDoT de-cellularised organs, Table S3: Comparison of proteomics analysis of ISDoT Lung vs Mammary and Fibroblast ECM Lung vs Mammary, Table S4: Protein Kinase Inhibitor Libraries stock plates used for drug screening on FDM’s, Table S5: Drug Inhibition of 4T1 cells on Plastic, Lung FDM, Gelatin, Matrigel and mCAF1 FDM compared to DMSO control, Table S6: Tyrosine peptide analysis of breast cancer cell responses to fibroblast-derived matrices, Serine and threonine peptide analysis of breast cancer cell responses to fibroblast-derived matrices, Table S7: Tyrosine peptide analysis of breast cancer responses to fibroblast-derived matrices, Serine/Threonine analysis of breast cancer responses to fibroblast-derived matrices.

## References

[1] Hanahan D., Weinberg R.A. (2000). The hallmarks of cancer. Cell.

[2] Dillekås H., Rogers M.S., Straume O. (2019). Are 90% of deaths from cancer caused by metastases?. Cancer Med..

[3] Cox T.R., Erler J.T. (2011). Remodeling and homeostasis of the extracellular matrix: Implications for fibrotic diseases and cancer. Dis. Models Mech..

[4] Cox T.R. (2021). The matrix in cancer. Nat. Rev. Cancer.

[5] Langley R.R., Fidler I.J. (2011). The seed and soil hypothesis revisited—The role of tumor-stroma interactions in metastasis to different organs. Int. J. Cancer.

[6] Kalluri R. (2016). The biology and function of fibroblasts in cancer. Nat. Rev. Cancer.

[7] Sahai E., Astsaturov I., Cukierman E., DeNardo D.G., Egeblad M., Evans R.M., Fearon D., Greten F.R., Hingorani S.R., Hunter T. (2020). A framework for advancing our understanding of cancer-associated fibroblasts. Nat. Rev. Cancer.

[8] Burgstaller G., Oehrle B., Gerckens M., White E.S., Schiller H.B., Eickelberg O. (2017). The instructive extracellular matrix of the lung: Basic composition and alterations in chronic lung disease. Eur. Respir. J..

[9] Lee J.Y., Chang J.K., Dominguez A.A., Lee H.P., Nam S., Chang J., Varma S., Qi L.S., West R.B., Chaudhuri O. (2019). YAP-independent mechanotransduction drives breast cancer progression. Nat. Commun..

[10] Brett E., Sauter M., Timmins É., Azimzadeh O., Rosemann M., Merl-Pham J., Hauck S.M., Nelson P.J., Becker K.F., Schunn I. (2020). Oncogenic Linear Collagen VI of Invasive Breast Cancer Is Induced by CCL5. J. Clin. Med..

[11] Park D., Sahai E., Rullan A. (2020). SnapShot: Cancer-Associated Fibroblasts. Cell.

[12] Naba A., Clauser K.R., Hoersch S., Liu H., Carr S.A., Hynes R.O. (2012). The matrisome: In silico definition and in vivo characterization by proteomics of normal and tumor extracellular matrices. Mol. Cell. Proteom..

[13] Kaukonen R., Jacquemet G., Hamidi H., Ivaska J. (2017). Cell-derived matrices for studying cell proliferation and directional migration in a complex 3D microenvironment. Nat. Protoc..

[14] Qi L., Zhang Y., Song F., Han Y., Ding Y. (2021). A newly identified small molecular compound acts as a protein kinase inhibitor to suppress metastasis of colorectal cancer. Bioorg. Chem..

[15] Manning G., Whyte D.B., Martinez R., Hunter T., Sudarsanam S. (2002). The protein kinase complement of the human genome. Science.

[16] Tan H.-Y., Wang N., Lam W., Guo W., Feng Y., Cheng Y.-C. (2018). Targeting tumour microenvironment by tyrosine kinase inhibitor. Mol. Cancer.

[17] Du Z., Lovly C.M. (2018). Mechanisms of receptor tyrosine kinase activation in cancer. Mol. Cancer.

[18] Heldin C.H. (2013). Targeting the PDGF signaling pathway in tumor treatment. Cell Commun. Signal..

[19] Normanno N., De Luca A., Bianco C., Strizzi L., Mancino M., Maiello M.R., Carotenuto A., De Feo G., Caponigro F., Salomon D.S. (2006). Epidermal growth factor receptor (EGFR) signaling in cancer. Gene.

[20] Roskoski R. (2020). Properties of FDA-approved small molecule protein kinase inhibitors: A 2020 update. Pharmacol. Res..

[21] Ediriweera M.K., Tennekoon K.H., Samarakoon S.R. (2019). In vitro assays and techniques utilized in anticancer drug discovery. J. Appl. Toxicol..

[22] Driehuis E., Kretzschmar K., Clevers H. (2020). Establishment of patient-derived cancer organoids for drug-screening applications. Nat. Protoc..

[23] Kitaeva K.V., Rutland C.S., Rizvanov A.A., Solovyeva V.V. (2020). Cell Culture Based in vitro Test Systems for Anticancer Drug Screening. Front. Bioeng. Biotechnol..

[24] Press D.J., Miller M.E., Liederbach E., Yao K., Huo D. (2017). De novo metastasis in breast cancer: Occurrence and overall survival stratified by molecular subtype. Clin. Exp. Metastasis.

[25] Liu J., Pandya P., Afshar S. (2021). Therapeutic Advances in Oncology. Int. J. Mol. Sci..

[26] Waks A.G., Winer E.P. (2019). Breast Cancer Treatment: A Review. JAMA.

[27] Aslakson C.J., Miller F.R. (1992). Selective events in the metastatic process defined by analysis of the sequential dissemination of subpopulations of a mouse mammary tumor. Cancer Res..

[28] Pulaski B.A., Ostrand-Rosenberg S. (2001). Mouse 4T1 breast tumor model. Curr. Protoc. Immunol..

[29] Franco-Barraza J., Beacham D.A., Amatangelo M.D., Cukierman E. (2016). Preparation of Extracellular Matrices Produced by Cultured and Primary Fibroblasts. Curr. Protoc. Cell Biol..

[30] Schoof E.M., Lechman E.R., Dick J.E. (2016). Global proteomics dataset of miR-126 overexpression in acute myeloid leukemia. Data Brief.

[31] Rappsilber J., Mann M., Ishihama Y. (2007). Protocol for micro-purification, enrichment, pre-fractionation and storage of peptides for proteomics using StageTips. Nat. Protoc..

[32] Mayorca-Guiliani A.E., Madsen C.D., Cox T.R., Horton E.R., Venning F.A., Erler J.T. (2017). ISDoT: In situ decellularization of tissues for high-resolution imaging and proteomic analysis of native extracellular matrix. Nat. Med..

[33] Hurkmans D.P., Verdegaal E.M.E., Hogan S.A., de Wijn R., Hovestad L., van den Heuvel D.M.A., Ruijtenbeek R., Welters M.J.P., van Brakel M., Basak E.A. (2020). Blood-based kinase activity profiling: A potential predictor of response to immune checkpoint inhibition in metastatic cancer. J. ImmunoTher. Cancer.

[34] Blache U., Horton E.R., Xia T., Schoof E.M., Blicher L.H., Schönenberger A., Snedeker J.G., Martin I., Erler J.T., Ehrbar M. (2019). Mesenchymal stromal cell activation by breast cancer secretomes in bioengineered 3D microenvironments. Life Sci. Alliance.

[35] Cline M.S., Smoot M., Cerami E., Kuchinsky A., Landys N., Workman C., Christmas R., Avila-Campilo I., Creech M., Gross B. (2007). Integration of biological networks and gene expression data using Cytoscape. Nat. Protoc..

[36] Warde-Farley D., Donaldson S.L., Comes O., Zuberi K., Badrawi R., Chao P., Franz M., Grouios C., Kazi F., Lopes C.T. (2010). The GeneMANIA prediction server: Biological network integration for gene prioritization and predicting gene function. Nucleic Acids Res..

[37] Horton E.R. (2021). Functional Bioinformatics Analyses of the Matrisome and Integrin Adhesome. Methods Mol. Biol..

[38] Wiese R., Eiglsperger M., Kaufmann M., Jünger M., Mutzel P. (2004). yFiles—Visualization and Automatic Layout of Graphs. Graph Drawing Software.

[39] Schwill M., Tamaskovic R., Gajadhar A.S., Kast F., White F.M., Plückthun A. (2019). Systemic analysis of tyrosine kinase signaling reveals a common adaptive response program in a HER2-positive breast cancer. Sci. Signal..

[40] Primac I., Maquoi E., Blacher S., Heljasvaara R., Van Deun J., Smeland H.Y.H., Canale A., Louis T., Stuhr L., Sounni N.E. (2019). Stromal integrin α11 regulates PDGFRβ signaling and promotes breast cancer progression. J. Clin. Investig..

[41] Perez-Garcia J., Muñoz-Couselo E., Soberino J., Racca F., Cortes J. (2018). Targeting FGFR pathway in breast cancer. Breast.

[42] Rane M.J., Coxon P.Y., Powell D.W., Webster R., Klein J.B., Pierce W., Ping P., McLeish K.R. (2001). p38 Kinase-dependent MAPKAPK-2 activation functions as 3-phosphoinositide-dependent kinase-2 for Akt in human neutrophils. J. Biol. Chem.

[43] Horiuchi D., Camarda R., Zhou A.Y., Yau C., Momcilovic O., Balakrishnan S., Corella A.N., Eyob H., Kessenbrock K., Lawson D.A. (2016). PIM1 kinase inhibition as a targeted therapy against triple-negative breast tumors with elevated MYC expression. Nat. Med..

[44] McLaughlin R.P., He J., van der Noord V.E., Redel J., Foekens J.A., Martens J.W.M., Smid M., Zhang Y., van de Water B. (2019). A kinase inhibitor screen identifies a dual cdc7/CDK9 inhibitor to sensitise triple-negative breast cancer to EGFR-targeted therapy. Breast Cancer Res..

[45] Margue C., Philippidou D., Kozar I., Cesi G., Felten P., Kulms D., Letellier E., Haan C., Kreis S. (2019). Kinase inhibitor library screening identifies synergistic drug combinations effective in sensitive and resistant melanoma cells. J. Exp. Clin. Cancer Res..

[46] Araujo J., Logothetis C. (2010). Dasatinib: A potent SRC inhibitor in clinical development for the treatment of solid tumors. Cancer Treat. Rev..

[47] Pietras K., Östman A. (2010). Hallmarks of cancer: Interactions with the tumor stroma. Exp. Cell Res..

[48] Calvo F., Ege N., Grande-Garcia A., Hooper S., Jenkins R.P., Chaudhry S.I., Harrington K., Williamson P., Moeendarbary E., Charras G. (2013). Mechanotransduction and YAP-dependent matrix remodelling is required for the generation and maintenance of cancer-associated fibroblasts. Nat. Cell Biol..

[49] Fidler I.J. (2003). The pathogenesis of cancer metastasis: The ‘seed and soil’ hypothesis revisited. Nat. Rev. Cancer.

[50] Rafaeva M., Erler J.T. (2020). Framing cancer progression: Influence of the organ- and tumour-specific matrisome. FEBS J..

[51] Shinsato Y., Doyle A.D., Li W., Yamada K.M. (2020). Direct comparison of five different 3D extracellular matrix model systems for characterization of cancer cell migration. Cancer Rep..

[52] Hutchinson L., Kirk R. (2011). High drug attrition rates—where are we going wrong?. Nat. Rev. Clin. Oncol..

[53] Yemanyi F., Vranka J., Raghunathan V., Caballero D., Kundu S.C., Reis R.L. (2020). Chapter 12—Generating cell-derived matrices from human trabecular meshwork cell cultures for mechanistic studies. Methods in Cell Biology.

[54] Walker C., Mojares E., Del Río Hernández A. (2018). Role of Extracellular Matrix in Development and Cancer Progression. Int. J. Mol. Sci..

[55] Pickup M.W., Laklai H., Acerbi I., Owens P., Gorska A.E., Chytil A., Aakre M., Weaver V.M., Moses H.L. (2013). Stromally derived lysyl oxidase promotes metastasis of transforming growth factor-β-deficient mouse mammary carcinomas. Cancer Res..

[56] Kutys M.L., Doyle A.D., Yamada K.M. (2013). Regulation of cell adhesion and migration by cell-derived matrices. Exp. Cell Res..

[57] Petrie R.J., Gavara N., Chadwick R.S., Yamada K.M. (2012). Nonpolarized signaling reveals two distinct modes of 3D cell migration. J. Cell Biol..

[58] Mazhar S., Taylor S.E., Sangodkar J., Narla G. (2019). Targeting PP2A in cancer: Combination therapies. Biochim. Biophys. Acta Mol. Cell Res..

[59] Kim J., Koo B.-K., Knoblich J.A. (2020). Human organoids: Model systems for human biology and medicine. Nat. Rev. Mol. Cell Biol..

[60] Maru Y., Tanaka N., Itami M., Hippo Y. (2019). Efficient use of patient-derived organoids as a preclinical model for gynecologic tumors. Gynecol. Oncol..

[61] Li Z., Dranoff J.A., Chan E.P., Uemura M., Sévigny J., Wells R.G. (2007). Transforming growth factor-beta and substrate stiffness regulate portal fibroblast activation in culture. Hepatology.

[62] Hoshiba T., Tanaka M. (2016). Decellularized matrices as in vitro models of extracellular matrix in tumor tissues at different malignant levels: Mechanism of 5-fluorouracil resistance in colorectal tumor cells. Biochim. Biophys. Acta BBA Mol. Cell Res..

