# Supplementary material for: Organ-Specific, Fibroblast-Derived Matrix as a Tool for Studying Breast Cancer Metastasis
Source: Cancers (Basel). 2021 Jul 2;13(13):3331. doi: 10.3390/cancers13133331 (PMC8269313; doi:10.3390/cancers13133331)
Supplement: Supplementary file 1 [file cancers-13-03331-s001.zip › cancers-1231266-supplementary.pdf]

cancers

MDPI

Organ-Specific, Fibroblast-Derived Matrix as a Tool for Studying Breast Cancer Metastasis

Adina R. D. Jensen, Edward R. Horton, Lene H. Blicher, Elin J. Pietras, Cornelia Steinhauer, Raphael Reuten, Erwin M. Schoof and Janine T. Erler

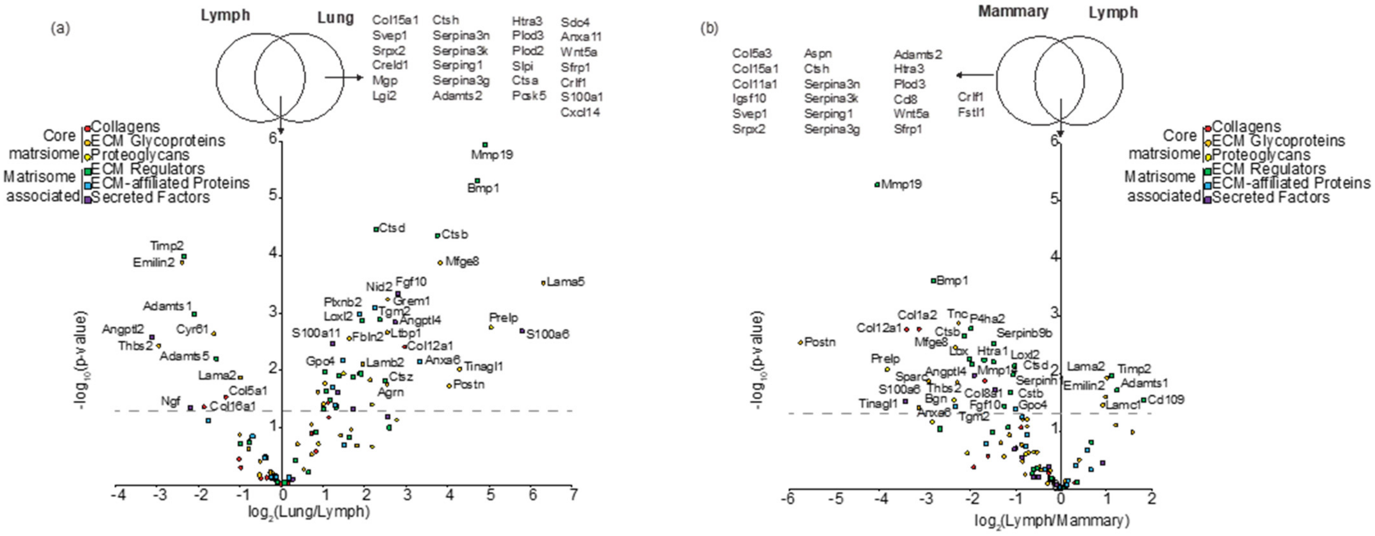

Figure S1. (**A**) Results of quantitative mass spectrometry, filtered for matrisome, of lymph vs lung fibroblast derived matrix (−log10 *p*-value 1.3 indicates proteins significantly higher in each condition). Venn diagram displays which lung and lymph ECM proteins were only detected in the lung ECM. (**B**) Results of quantitative mass spectrometry, filtered for matrisome, of mammary vs lymph fibroblast derived matrix (−log10 *p*-value 1.3 indicates proteins significantly higher in each condition). Venn diagram displays which lymph and mammary ECM proteins were only detected in the mammary ECM.

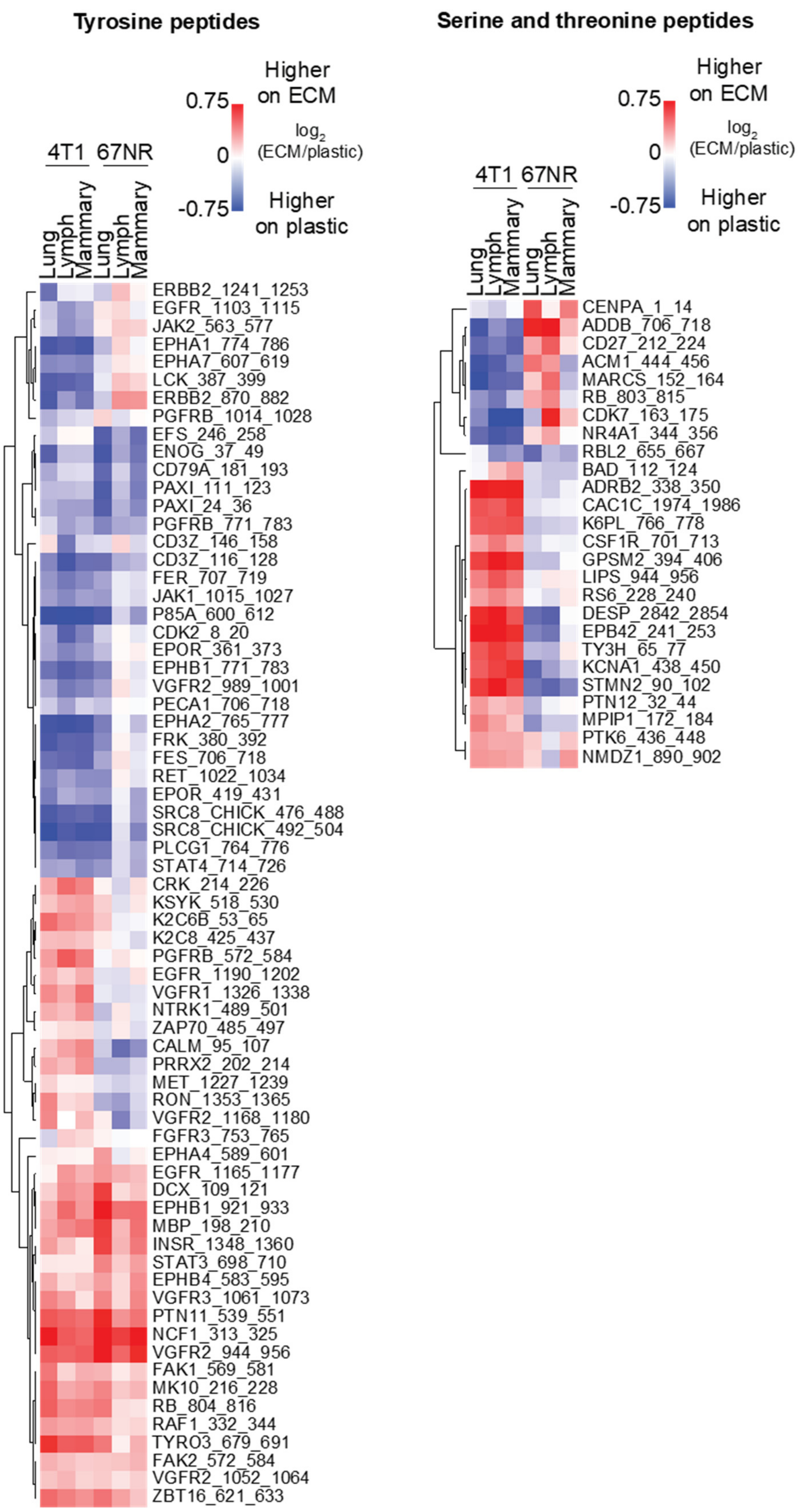

Figure S2. Hierarchical clustering of tyrosine and serine/threonine peptides of 4T1 and 67NR cells on lung, lymph and mammary FDMs compared to plastic. Red indicates peptides more phosphorylated on the ECM. Blue indicates peptides more phosphorylated on plastic.

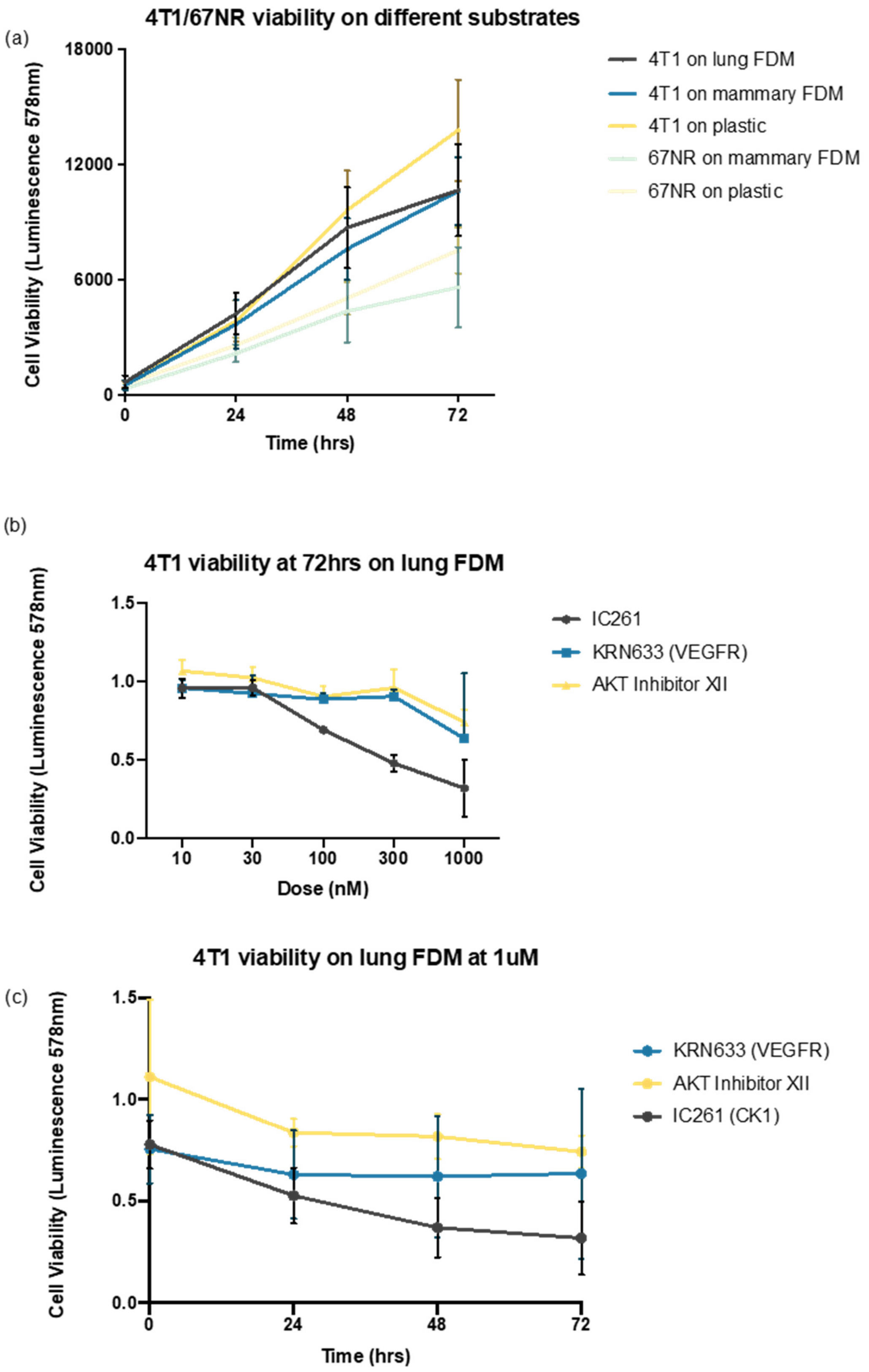

Figure S3. (**a**) Cell viability of 4T1 and 67NR cells on different substrates (lung, mammary and plastic). (**b**) Cell viability of 4T1 cells (72 hrs) when treated with 3 compounds which had previously reduced 4T1 proliferation on lung FDM (drug screen). (**c**) Dose response of 4T1 viability for 3 compounds (at 1 uM).

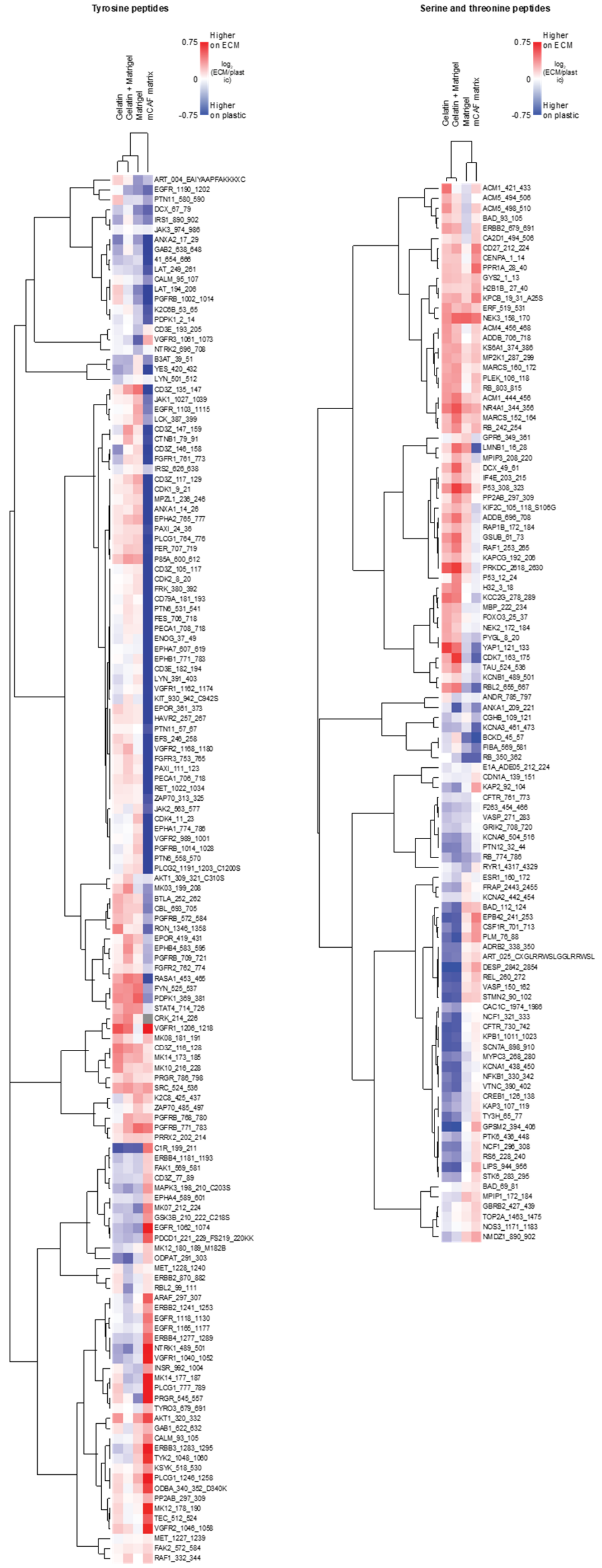

Figure S4. Hierarchical clustering of tyrosine and serine/threonine peptides of 4T1 cells on gelatin, gelatin + Matrigel, Matrigel and mCAF1 FDM compared to plastic. Red indicates peptides more phosphorylated on the ECM. Blue indicates peptides more phosphorylated on plastic.
